# Cytological evaluation of breast lesions in symptomatic patients presenting to Kenyatta National Hospital, Kenya: a retrospective study

**DOI:** 10.1186/s12905-015-0278-y

**Published:** 2015-12-15

**Authors:** Ken Munene Nkonge, Emily Adhiambo Rogena, Edwin Owino Walong, Dennis Karani Nkonge

**Affiliations:** School of Medicine, University of Nairobi, P.O. Box 19676, Nairobi, Kenya; Department of Human Pathology, School of Medicine, University of Nairobi, P.O. Box 19676, Nairobi, Kenya

**Keywords:** Breast, Cytopathology, Fibroadenoma, Gynecomastia, Synoptic reporting

## Abstract

**Background:**

Palpable breast lump, breast pain, and nipple discharge are common symptoms of breast disease. Breast cytology (fine-needle aspiration, nipple discharge smear, and touch preparation) accurately identifies benign, atypical, and malignant pathological changes in breast specimens. This study aims to determine the types of breast lesions diagnosed by breast cytology and assess the clinical adequacy of narrative reporting of breast cytology results.

**Methods:**

Medical records of 390 patients presenting to breast or general surgery clinics in Kenyatta National Hospital, Nairobi, Kenya, between January 2010 and March 2014 were evaluated retrospectively.

**Results:**

Of the 390 diagnosed breast lesions, 89.7 % (*n* = 350) occurred in females, while 10.3 % (*n* = 40) occurred in males, giving rise to a female-to-male ratio of 8.8:1. Neoplastic breast lesions (*n* = 296) comprised 75.9 %, while non-neoplastic breast lesions (*n* = 94) comprised 24.1 % of all diagnosed breast lesions. The neoplastic lesions were classified as 72.3 % (*n* = 214) benign and 27.7 % (*n* = 82) malignant, resulting in a benign-to-malignant ratio of 2.6:1. Fibroadenoma (*n* = 136) and gynecomastia (*n* = 33) were the most frequently diagnosed breast lesions for women and men, respectively.

**Conclusions:**

Breast cytology effectively diagnosed neoplastic and non-neoplastic breast lesions. Neoplastic breast lesions occurred more frequently in women whereas non-neoplastic lesions occurred more frequently in men. To address the limitations associated with narrative reporting of breast cytology results, a synoptic reporting format incorporating the United Kingdom’s National Health Service Breast Screening Programme’s diagnostic categories (C1 to C5) is recommended for adoption by this hospital.

## Background

Palpable breast lump, breast pain, and nipple discharge are common manifestations of benign, premalignant, or malignant lesions in the human mammary gland and surrounding tissues [1–4]. Techniques used to diagnose breast lesions include clinical breast examination, breast imaging, and breast cytology [2, 4]. Fine-needle aspiration cytology is the most reliable component of this triple test assessment of breast lesions due to its high sensitivity, specificity, negative predictive value, and positive predictive value [3, 5].

Malignant and benign breast diseases are prevalent in girls and women in Sub-Saharan Africa [6, 7] as exemplified by three studies from Kenyatta National hospital (KNH), a tertiary referral and teaching hospital in Nairobi, Kenya. A histologic study of 1501 breast specimens conducted by Bjerregaard and Kung’u found that benign and malignant lesions accounted for 72.2 % and 27.8 % of the diagnosed breast lesions, respectively [8]. A retrospective study of 1172 patient records performed by Otieno et al. found that fibroadenoma (33.2 %) and ductal carcinoma (17.4 %) were the most frequently diagnosed types of lesions and 98.9 % of all breast lesions occurred in female patients [9]. Finally, a prospective cross-sectional study of 166 breast cancer patients conducted by Otieno et al. found that 98.8 % of patients diagnosed with breast cancer were females and 24.1 % of patients had been incorrectly reassured that their disease was benign prior to being diagnosed with breast cancer [10].

The primary objective of this study was to determine the type and sex-specific distribution of breast lesions diagnosed by cytological evaluation of breast specimens from patients presenting to KNH with breast complaints over a four year period. The secondary objective of this study was to evaluate the clinical adequacy of narrative reporting of breast cytology results.

## Methods

Medical records in the form of breast cytology reports from 443 consecutive patients presenting to breast or general surgical clinics at KNH over the period of January 5, 2010 and March 6, 2014 with palpable breast lump, nipple discharge, breast pain, nipple retraction, skin changes, or axillary lymphadenopathy were accessed from the records department of KNH’s cytology laboratory and examined for eligibility. Reports having patient age and sex, clinical summary, breast cytology sampling technique, microscopic findings, and conclusive breast cytology diagnosis were included in the study. Reports having major typographical errors, clinical history of breast cancer, cytological diagnosis of secondary breast diseases, and inconclusive breast cytology results were excluded from the study. A total of 390 breast cytology reports satisfied eligibility criteria. All data were analyzed using Microsoft Excel 2013 and results described using summary statistics. This single institution study was approved by the Kenyatta National Hospital/University of Nairobi Ethics and Research Committee (Ref: KNH-ERC/UA/189). The need for written or verbal informed consent was waived by the KNH-ERC.

## Results

### Presenting complaints and diagnosed breast lesions

The mean age of the evaluated patients was 36.0 ± 16.7 (range, 10–90) years and the sex-specific mean age was 34.6 ± 16.2 (range, 10–90) years for women and 48.6 ± 16.2 (range, 15–78) years for men. Breast lesions were most frequently diagnosed in women aged 20–24 years (*n* = 91) and 50+ years (*n* = 65), and men aged 50+ years (*n* = 25). The presenting complaints are summarized in Table 1. Palpable breast lump was the most common (96.7 %; *n* = 377) while nipple retraction was the least common (1.3 %; *n* = 5) presenting complaint. The median duration of presenting complaints in patients was 11 months for women and 4.5 months for men.

**Table 1:** Presenting complaints of study population (*n* = 390)

Presenting complaint	Women	Men	Total
Palpable breast lump	337	40	377
Nipple discharge	29	0	29
Breast pain	19	8	27
Skin changes	22	0	22
Palpable axillary lymph node(s)	15	0	15
Nipple retraction	5	0	5
Median duration (in months)	11	4.5	8

**Fig. 1:**
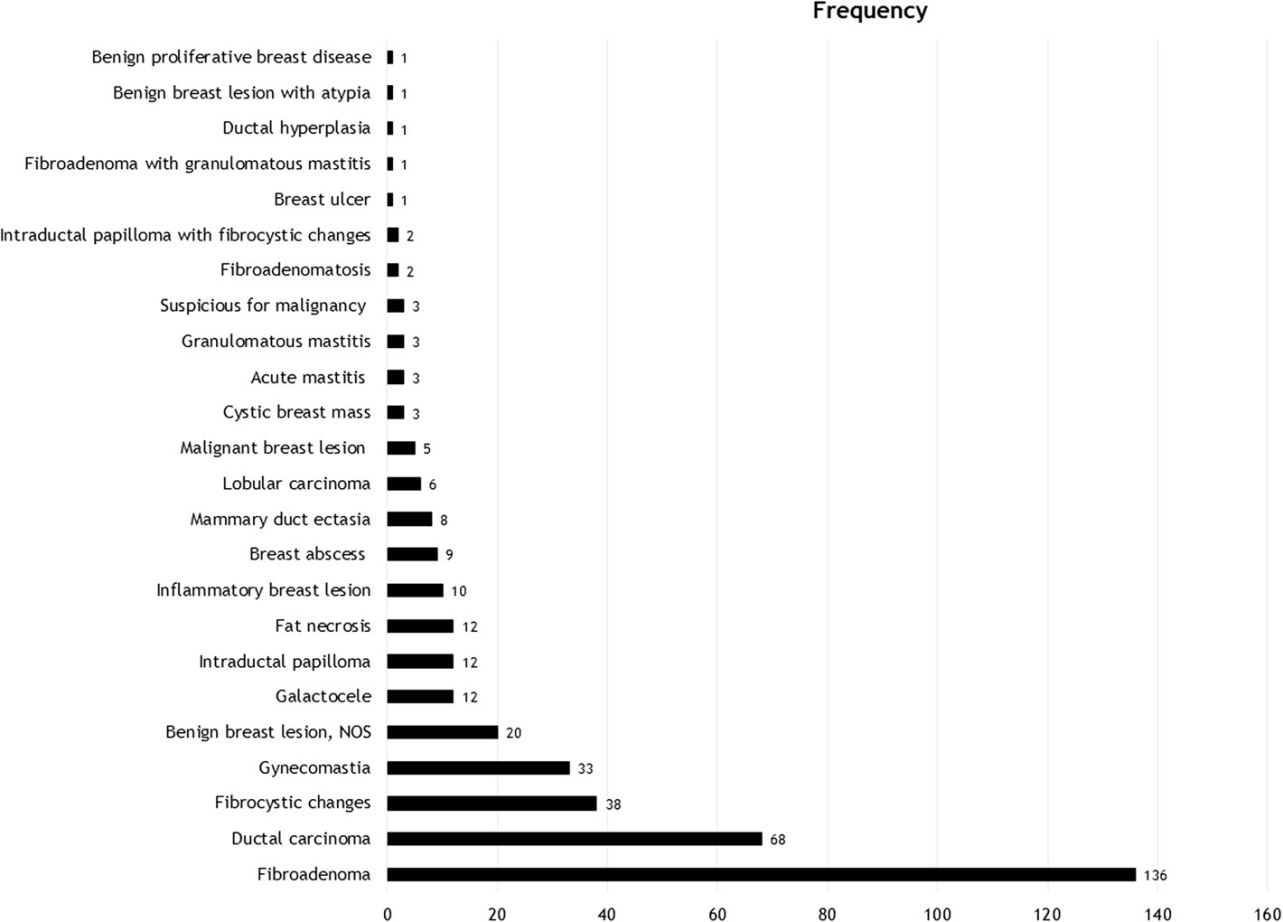
Cytological profile of breast lesions diagnosed in women and men (*n* = 390). Bar chart represents the frequency distribution of all breast lesions diagnosed from January 2010 to March 2014. NOS: not otherwise specified

The frequency distribution of all diagnosed breast lesions is shown in Fig. 1. Fibroadenoma (*n* = 136) and fibrocystic changes (*n* = 38) were the most frequently diagnosed benign breast lesions whereas ductal carcinoma (*n* = 68) was the most frequently diagnosed malignant breast lesion. Other frequently diagnosed breast lesions included gynecomastia (*n* = 33), benign breast lesions, not otherwise specified (*n* = 20), galactocele (*n* = 12), intraductal papilloma (*n* = 12), and fat necrosis (*n* = 12).

### Types of diagnosed breast lesions and sex-specific distribution

Of the 390 diagnosed breast lesions, 75.9 % (*n* = 296) were neoplastic and 24.1 % (*n* = 94) were non-neoplastic. Of the neoplastic breast lesions, 72.3 % (*n* = 214) were benign and 27.7 % (*n* = 82) were malignant. Thus, the benign-to-malignant ratio was 2.6:1. Of the non-neoplastic breast lesions, 48.9 % (*n* = 46) were associated with inflammation whereas 51.1 % (*n* = 48) were not associated with inflammation.

The sex-specific distribution of breast lesions diagnosed by breast cytology (Table 2) shows that 98 % of the neoplastic and 63.8 % of the non-neoplastic breast lesions occurred in women. Fibroadenoma (38.9 %), ductal carcinoma (18 %), and fibrocystic changes (10.9 %) were the most frequently diagnosed breast lesions in women. Fibroadenoma affected women aged 10–57 years, ductal carcinoma affected women aged 21–90 years, and fibrocystic changes affected women aged 21–67 years. Lobular carcinoma affected women aged 37–83 years and accounted for 1.7 % of the breast lesions diagnosed in women.

**Table 2:** Sex-specific distribution of breast lesions diagnosed by breast cytology

Cytological diagnoses	Women	Men	Total
	n (%)	n (%)	n (%)
*A. Neoplastic*			
Ductal carcinoma	63 (18 %)	5 (12.5 %)	68 (17.4 %)
Fibroadenoma	136 (38.9 %)	0 (0 %)	136 (34.9 %)
Fibrocystic changes	38 (10.9 %)	0 (0 %)	38 (9.7 %)
Benign breast lesion, NOS	19 (5.4 %)	1 (2.5 %)	20 (5.1 %)
Intraductal papilloma	12 (3.4 %)	0 (0 %)	12 (3.1 %)
Lobular carcinoma	6 (1.7 %)	0 (0 %)	6 (1.5 %)
Malignant breast lesion	5 (1.4 %)	0 (0 %)	5 (1.3 %)
Suspicious for malignancy	3 (0.9 %)	0 (0 %)	3 (0.8 %)
Fibroadenomatosis	2 (0.6 %)	0 (0 %)	2 (0.5 %)
Intraductal papilloma with fibrocystic changes	2 (0.6 %)	0 (0 %)	2 (0.5 %)
Fibroadenoma with granulomatous mastitis	1 (0.3 %)	0 (0 %)	1 (0.3 %)
Ductal hyperplasia	1 (0.3 %)	0 (0 %)	1 (0.3 %)
Benign breast lesion with atypia	1 (0.3 %)	0 (0 %)	1 (0.3 %)
Benign proliferative breast disease	1 (0.3 %)	0 (0 %)	1 (0.3 %)
*B. Non-neoplastic*			
Gynecomastia	0 (0 %)	33 (82.5 %)	33 (8.5 %)
Galactocele	12 (3.4 %)	0 (0 %)	12 (3.1 %)
Fat necrosis	12 (3.4 %)	0 (0 %)	12 (3.1 %)
Breast abscess	9 (2.6 %)	0 (0 %)	9 (2.3 %)
Inflammatory breast lesion	9 (2.6 %)	1 (2.5 %)	10 (2.6 %)
Mammary duct ectasia	8 (2.3 %)	0 (0 %)	8 (2.1 %)
Acute mastitis	3 (0.9 %)	0 (0 %)	3 (0.8 %)
Cystic breast mass	3 (0.9 %)	0 (0 %)	3 (0.8 %)
Granulomatous mastitis	3 (0.9 %)	0 (0 %)	3 (0.8 %)
Breast ulcer	1 (0.3 %)	0 (0 %)	1 (0.3 %)

In men, 2 % of the breast lesions were neoplastic whereas 36.2 % were non-neoplastic. Gynecomastia (82.5 %) was the most frequent breast lesion in men and was diagnosed in men aged 17–67 years. Ductal carcinoma (12.5 %) was the only neoplastic breast lesion and was diagnosed in men aged 52–68 years. Taken together, 89.7 % (*n* = 350) of breast lesions occurred in females, while 10.3 % (*n* = 40) of breast lesions occurred in males, giving rise to a female-to-male ratio of 8.8:1.

### Clinical adequacy of narrative reporting of breast cytology results

**Fig. 2:**
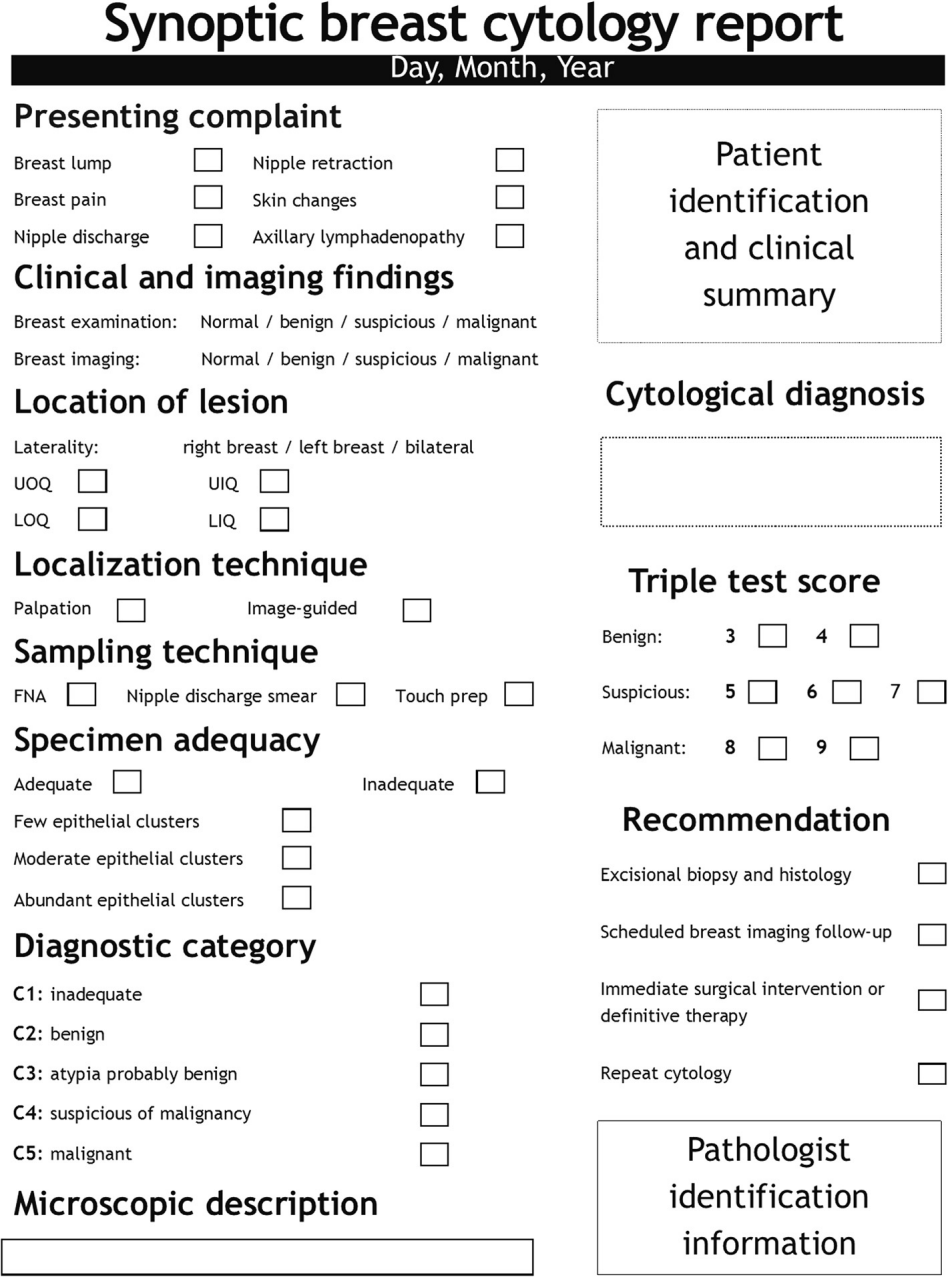
Recommended synoptic reporting format for recording breast cytology results. The recommended synoptic reporting format consists of clinically relevant parameters in checklist form with minimal use of narrative and incorporates standardized diagnostic terminology. FNA: fine-needle aspiration; LIQ: lower inner quadrant; LOQ: lower outer quadrant; UIQ: upper inner quadrant; UOQ: upper outer quadrant

Results from the 390 breast cytology reports evaluated were reported in descriptive narrative format. Based on the findings from Abati and Simsir’s review [11] of the 1996 National Cancer Institute Consensus Panel Criteria for optimal performance and reporting of breast fine-needle aspiration [12], breast cytology reports from KNH were found clinically inadequate and requiring improvement. All the reports lacked standardized diagnostic terminology and did not clearly report clinical and imaging findings, lesion localization technique, specimen adequacy, triple test assessment findings, and post-cytology recommendations. To address these limitations, a synoptic reporting format (Fig. 2) has been developed and is recommended for adoption by KNH. The recommended synoptic reporting format clearly shows the clinical and imaging findings, lesion localization technique, triple test score, and post-cytology recommendations and also incorporates the National Health Service Breast Screening Programme’s (NHSBSP) breast cytology diagnostic categories: inadequate (C1), benign (C2), atypia probably benign (C3), suspicious of malignancy (C4), and malignant (C5) [13, 14]. Notably, integration of triple test findings to the recommended synoptic reporting format is expected to facilitate clinical decision-making by stratifying patients into three evidence-based groups [4, 15]: patients eligible for scheduled breast imaging follow-up based on triple test scores of 3–4, patients eligible for excisional biopsy and cytohistologic correlation based on triple test scores of 5–7, and patients eligible for immediate surgical intervention or definitive therapy based on triple test scores of 8–9 and overall clinical impression.

## Discussion

The primary objective of the present study was to determine the type and distribution of breast lesions diagnosed by breast cytology in Kenyan patients presenting to a tertiary referral hospital over a four year period. Of the total diagnosed breast lesions, 72.3 % were benign, which is in agreement with findings by Bjerregaard and Kung’u [8] and Panjvani et al. [16]. Fibroadenoma was the most frequently diagnosed lesion in pre-menopausal women, while gynecomastia was the most frequently diagnosed lesion in men, consistent with both prospective and retrospective studies from Uganda [7], India [16], and Pakistan [17]. Palpable breast lump (377 patients) was the most common presenting complaint, which is consistent with previous findings in Kenya [10] and elsewhere [2, 17, 18]. Nipple discharge (29 patients) was the second most common presenting complaint. Although nipple discharge is often suggestive of breast malignancy when unilateral, bloody, or associated with a breast lump [1, 19, 20], its diagnostic value is controversial [2, 19–22]. Consequently, more studies are needed to establish the suitability of triple test assessment for diagnostic evaluation of patients presenting with nipple discharge. Finally, despite the consistency of present findings with existing literature, the prevalence of breast diseases in Kenya may be underestimated by the retrospective study design. Population-based studies are therefore needed to fully characterize the epidemiology of breast diseases in Kenya.

The secondary objective of this study was to assess the clinical adequacy of narrative reporting of breast cytology results. All of the breast cytology reports evaluated in this study used a non-standardized descriptive narrative format instead of a standardized checklist-based synoptic format [23]. Synoptic reporting of pathology results is gaining acceptance in Asia and North America. A synoptic report was recently developed and used in Japan to accurately report breast cytology results in a series of 3439 cases [24]. Additionally, a cancer reporting project conducted in Ontario, Canada by Srigley et al. found that a province-wide implementation of a synoptic reporting format generated more complete reports and led to increased rates of cancer reporting [25]. Our findings alongside those from other investigators suggest that evidence-based synoptic reporting combined with active monitoring of results may be superior to traditional narrative reporting. Therefore, adoption of the recommended synoptic reporting format by KNH is expected to enhance the clinical utility of diagnostic breast cytology and promote standardization of breast pathology practices in Kenya.

## Conclusions

This study found that breast cytology effectively diagnosed neoplastic and non-neoplastic breast lesions. Neoplastic breast lesions occurred more frequently in women, while non-neoplastic lesions occurred more frequently in men. To address the limitations associated with narrative reporting of breast cytology results, KNH should adopt the recommended synoptic reporting format.

## Abbreviations

KNH: Kenyatta National Hospital
KNH-ERC: Kenyatta National Hospital/University of Nairobi Ethics and Research Committee
NHSBSP: National Health Service Breast Screening Programme
